# Ethyl 3-{[(3-methyl­anilino)(1*H*-1,2,4-triazol-1-yl)methyl­idene]amino}-1-benzofuran-2-carboxyl­ate

**DOI:** 10.1107/S1600536810042868

**Published:** 2010-10-30

**Authors:** Hai-Tao Gao, Li Li, Jun-Kai Ma

**Affiliations:** a Institute of Medicinal Chemistry, Hubei Medical University, Shiyan Hubei 442000, People’s Republic of China; b Hubei Medical University Library, Shiyan Hubei 442000, People’s Republic of China

## Abstract

The crystal structure of the title compound, C_21_H_19_N_5_O_3_, is stabilized by inter­molecular N—H⋯N and C—H⋯O hydrogen bonds. The mol­ecule contains a planar [maximum deviations = −0.026 (1) and 0.027 (2) Å] benzofuran ring system, which forms dihedral angles of 78.75 (8) and 39.78 (7)° with the benzene and triazole rings, respectively.

## Related literature

For the synthesis of heterocyclic compounds, see: Hu *et al.* (2007 ▶); Hu & Ding (2008 ▶). For related structures, see: Hu *et al.* (2010 ▶); Chen *et al.* (2008 ▶); Ma *et al.* (2009 ▶); Yang *et al.* (2009 ▶).
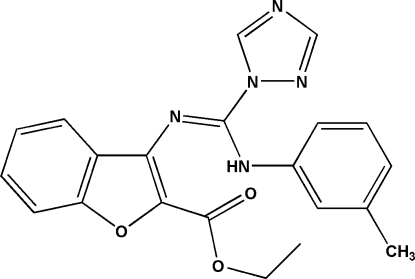


## Experimental

### 

#### Crystal data

C_21_H_19_N_5_O_3_
*M* *_r_* = 389.41
Monoclinic, 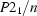

*a* = 10.967 (1) Å
*b* = 9.9606 (9) Å
*c* = 17.4807 (15) Å
β = 91.439 (1)°
*V* = 1909.0 (3) Å^3^
*Z* = 4
Mo *K*α radiation
μ = 0.09 mm^−1^
*T* = 298 K
0.30 × 0.20 × 0.10 mm

#### Data collection

Bruker SMART 4K CCD area-detector diffractometer
Absorption correction: multi-scan (*SADABS*; Sheldrick, 2003 ▶) *T*_min_ = 0.972, *T*_max_ = 0.991
14116 measured reflections
4713 independent reflections
3715 reflections with *I* > 2σ(*I*)
*R*_int_ = 0.031

#### Refinement

*R*[*F* ^2^ > 2σ(*F* ^2^)] = 0.054
*wR*(*F* ^2^) = 0.135
*S* = 1.07
4713 reflections
267 parameters
H atoms treated by a mixture of independent and constrained refinement
Δρ_max_ = 0.26 e Å^−3^
Δρ_min_ = −0.23 e Å^−3^

### 

Data collection: *SMART* (Bruker, 2001 ▶); cell refinement: *SAINT-Plus* (Bruker, 2001 ▶); data reduction: *SAINT-Plus*; program(s) used to solve structure: *SHELXS97* (Sheldrick, 2008 ▶); program(s) used to refine structure: *SHELXL97* (Sheldrick, 2008 ▶); molecular graphics: *PLATON* (Spek, 2009 ▶); software used to prepare material for publication: *SHELXTL* (Sheldrick, 2008 ▶).

## Supplementary Material

Crystal structure: contains datablocks I, global. DOI: 10.1107/S1600536810042868/jh2221sup1.cif

Structure factors: contains datablocks I. DOI: 10.1107/S1600536810042868/jh2221Isup2.hkl

Additional supplementary materials:  crystallographic information; 3D view; checkCIF report

## Figures and Tables

**Table 1:** Hydrogen-bond geometry (Å, °)

*D*—H⋯*A*	*D*—H	H⋯*A*	*D*⋯*A*	*D*—H⋯*A*
C10—H10⋯O2^i^	0.93	2.43	3.271 (2)	150
N1—H1⋯N5^ii^	0.862 (17)	2.250 (17)	3.0755 (19)	160.3 (15)

Symmetry codes: (i) 
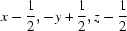
; (ii) 
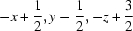
.

**Fig. 1.**
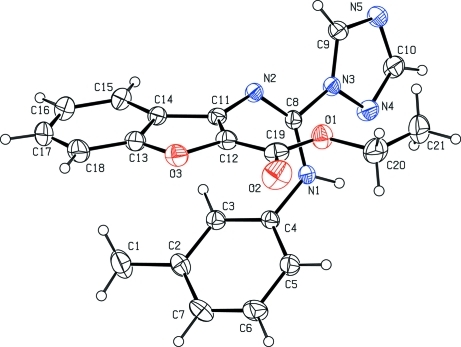
The molecular structure of the title compound, showing the atom-labeling scheme.

## supplementary crystallographic information

### Comment

As a part of our ongoing investigations on the preparation of derivatives of heterocyclic compounds (Hu *et al.*, 2007, 2008, 2010; Chen *et al.*, 2008; Ma *et al.*, 2009; Yang *et al.*, 2009), we have synthesized and structurally characterized characterized the title compound. Here we wish to report an *x*-ray crystal structure of it(Fig. 1). In the molecule, the mean plane of the benzofuran system make dihedral angle of 78.75 (8)°, 39.78 (7)°, with the phenyl(C2—C7) ring and the triazole ring, respectively. The crystal structure is mainly stabilized by weak intermolecular N—H···N and C—H···O hydrogen bonding interactions (Table. 1). There are no π-π interactions.

### Experimental

The title compound was obtained in excellent yield *via* aza-WIttig reaction. Crystals suitable for single-crystal X-ray diffraction were obtained by recrystallization from a mixed solvent of ethanol and dichloromethane (1:1 *v*/*v*) at room temperature.

### Refinement

All H-atoms were positioned with idealized geometry and refined isotropic (*U*_iso_(H)= 1.5*U*_eq_(C)for methyl H atoms and *U*_iso_(H) =1.2*U*_eq_(C) for all other H atoms) using a riding model with C—H = 0.93°, 0.97°, 0.96Å and N—H = 0.86°.

### Crystal data

**Table:**

C_21_H_19_N_5_O_3_	*F*(000) = 816
*M**_r_* = 389.41	*D*_x_ = 1.355 Mg m^−^^3^
Monoclinic, *P*2_1_/*n*	Mo *K*α radiation, λ = 0.71073 Å
Hall symbol: -P 2yn	Cell parameters from 4199 reflections
*a* = 10.967 (1) Å	θ = 2.2–25.7°
*b* = 9.9606 (9) Å	µ = 0.09 mm^−^^1^
*c* = 17.4807 (15) Å	*T* = 298 K
β = 91.439 (1)°	Block, colorless
*V* = 1909.0 (3) Å^3^	0.30 × 0.20 × 0.10 mm
*Z* = 4	

### Data collection

**Table:**

Bruker SMART 4K CCD area-detector diffractometer	4713 independent reflections
Radiation source: fine-focus sealed tube	3715 reflections with *I* > 2σ(*I*)
graphite	*R*_int_ = 0.031
φ and ω scans	θ_max_ = 28.3°, θ_min_ = 2.2°
Absorption correction: multi-scan (*SADABS*; Sheldrick, 2003)	*h* = −14→14
*T*_min_ = 0.972, *T*_max_ = 0.991	*k* = −13→12
14116 measured reflections	*l* = −23→22

### Refinement

**Table:**

Refinement on *F*^2^	Primary atom site location: structure-invariant direct methods
Least-squares matrix: full	Secondary atom site location: difference Fourier map
*R*[*F*^2^ > 2σ(*F*^2^)] = 0.054	Hydrogen site location: inferred from neighbouring sites
*wR*(*F*^2^) = 0.135	H atoms treated by a mixture of independent and constrained refinement
*S* = 1.07	*w* = 1/[σ^2^(*F*_o_^2^) + (0.0611*P*)^2^ + 0.263*P*] where *P* = (*F*_o_^2^ + 2*F*_c_^2^)/3
4713 reflections	(Δ/σ)_max_ = 0.001
267 parameters	Δρ_max_ = 0.26 e Å^−^^3^
0 restraints	Δρ_min_ = −0.23 e Å^−^^3^

### Special details

**Table:**

Geometry. All e.s.d.'s (except the e.s.d. in the dihedral angle between two l.s. planes) are estimated using the full covariance matrix. The cell e.s.d.'s are taken into account individually in the estimation of e.s.d.'s in distances, angles and torsion angles; correlations between e.s.d.'s in cell parameters are only used when they are defined by crystal symmetry. An approximate (isotropic) treatment of cell e.s.d.'s is used for estimating e.s.d.'s involving l.s. planes.
Refinement. Refinement of *F*^2^ against ALL reflections. The weighted *R*-factor *wR* and goodness of fit *S* are based on *F*^2^, conventional *R*-factors *R* are based on *F*, with *F* set to zero for negative *F*^2^. The threshold expression of *F*^2^ > σ(*F*^2^) is used only for calculating *R*-factors(gt) *etc*. and is not relevant to the choice of reflections for refinement. *R*-factors based on *F*^2^ are statistically about twice as large as those based on *F*, and *R*- factors based on ALL data will be even larger.

### Fractional atomic coordinates and isotropic or equivalent isotropic displacement parameters (Å^2^)

**Table:**

	*x*	*y*	*z*	*U*_iso_*/*U*_eq_	
C1	−0.0436 (2)	0.3180 (2)	1.13054 (12)	0.0700 (6)	
H1A	−0.0632	0.2688	1.1758	0.105*	
H1B	−0.1172	0.3376	1.1018	0.105*	
H1C	−0.0036	0.4003	1.1447	0.105*	
C2	0.03957 (14)	0.23527 (18)	1.08246 (9)	0.0430 (4)	
C3	0.09144 (14)	0.29052 (16)	1.01810 (8)	0.0365 (3)	
H3	0.0738	0.3790	1.0049	0.044*	
C4	0.16873 (13)	0.21673 (15)	0.97313 (8)	0.0322 (3)	
C5	0.19517 (16)	0.08510 (16)	0.99188 (9)	0.0448 (4)	
H5	0.2485	0.0354	0.9626	0.054*	
C6	0.14145 (18)	0.02809 (19)	1.05469 (11)	0.0558 (5)	
H6	0.1573	−0.0612	1.0670	0.067*	
C7	0.06476 (16)	0.10228 (19)	1.09921 (10)	0.0517 (5)	
H7	0.0293	0.0624	1.1413	0.062*	
C8	0.26228 (13)	0.39756 (14)	0.89881 (8)	0.0305 (3)	
C9	0.32102 (15)	0.54488 (16)	0.78959 (9)	0.0404 (4)	
H9	0.3687	0.6067	0.8167	0.048*	
C10	0.22640 (19)	0.44048 (17)	0.70420 (9)	0.0514 (5)	
H10	0.1958	0.4171	0.6559	0.062*	
C11	0.32117 (14)	0.45550 (15)	1.02485 (8)	0.0338 (3)	
C12	0.39857 (14)	0.36751 (16)	1.06072 (8)	0.0390 (4)	
C13	0.30913 (16)	0.47691 (17)	1.15284 (9)	0.0441 (4)	
C14	0.26388 (15)	0.53020 (16)	1.08480 (8)	0.0387 (4)	
C15	0.17586 (17)	0.63069 (17)	1.08694 (10)	0.0483 (4)	
H15	0.1442	0.6684	1.0420	0.058*	
C16	0.1368 (2)	0.6728 (2)	1.15721 (12)	0.0628 (5)	
H16	0.0776	0.7394	1.1596	0.075*	
C17	0.1844 (2)	0.6175 (2)	1.22463 (12)	0.0690 (6)	
H17	0.1569	0.6487	1.2713	0.083*	
C18	0.2710 (2)	0.5180 (2)	1.22393 (10)	0.0611 (5)	
H18	0.3024	0.4802	1.2689	0.073*	
C19	0.48201 (16)	0.26505 (17)	1.03424 (10)	0.0455 (4)	
C20	0.55170 (19)	0.1573 (2)	0.92310 (13)	0.0646 (6)	
H20A	0.5239	0.0695	0.9391	0.077*	
H20B	0.6372	0.1666	0.9377	0.077*	
C21	0.5346 (2)	0.1731 (2)	0.83896 (14)	0.0776 (7)	
H21A	0.4493	0.1680	0.8256	0.116*	
H21B	0.5774	0.1028	0.8133	0.116*	
H21C	0.5660	0.2587	0.8236	0.116*	
N1	0.21649 (12)	0.27264 (13)	0.90558 (7)	0.0353 (3)	
H1	0.2075 (15)	0.2264 (16)	0.8641 (10)	0.042*	
N2	0.30167 (12)	0.48370 (12)	0.94727 (7)	0.0344 (3)	
N3	0.26523 (11)	0.43926 (12)	0.82068 (7)	0.0333 (3)	
N4	0.20199 (15)	0.37086 (14)	0.76506 (7)	0.0493 (4)	
N5	0.29902 (14)	0.54902 (14)	0.71559 (7)	0.0480 (4)	
O1	0.48087 (11)	0.26108 (12)	0.95828 (7)	0.0489 (3)	
O2	0.54273 (14)	0.19350 (14)	1.07513 (8)	0.0728 (4)	
O3	0.39307 (11)	0.37876 (12)	1.13978 (6)	0.0483 (3)	

### Atomic displacement parameters (Å^2^)

**Table:**

	*U*^11^	*U*^22^	*U*^33^	*U*^12^	*U*^13^	*U*^23^
C1	0.0739 (14)	0.0835 (15)	0.0539 (12)	0.0117 (12)	0.0255 (11)	0.0014 (11)
C2	0.0384 (8)	0.0575 (10)	0.0330 (8)	−0.0004 (7)	0.0014 (7)	0.0014 (7)
C3	0.0376 (8)	0.0376 (8)	0.0342 (8)	0.0000 (6)	−0.0007 (6)	0.0019 (6)
C4	0.0355 (7)	0.0366 (8)	0.0244 (7)	−0.0050 (6)	−0.0027 (6)	0.0015 (6)
C5	0.0550 (10)	0.0399 (9)	0.0396 (9)	0.0056 (8)	0.0063 (7)	0.0032 (7)
C6	0.0690 (12)	0.0441 (10)	0.0545 (11)	0.0053 (9)	0.0079 (9)	0.0184 (8)
C7	0.0506 (10)	0.0657 (12)	0.0391 (9)	−0.0011 (9)	0.0075 (8)	0.0184 (9)
C8	0.0350 (7)	0.0313 (7)	0.0251 (7)	0.0066 (6)	0.0005 (5)	0.0022 (6)
C9	0.0506 (9)	0.0391 (8)	0.0315 (8)	−0.0036 (7)	0.0015 (7)	0.0032 (6)
C10	0.0851 (13)	0.0421 (9)	0.0266 (8)	−0.0038 (9)	−0.0074 (8)	0.0010 (7)
C11	0.0397 (8)	0.0327 (7)	0.0288 (7)	−0.0079 (6)	−0.0026 (6)	0.0006 (6)
C12	0.0433 (8)	0.0432 (9)	0.0299 (8)	−0.0055 (7)	−0.0073 (6)	0.0053 (6)
C13	0.0548 (10)	0.0458 (9)	0.0314 (8)	−0.0127 (8)	−0.0031 (7)	−0.0007 (7)
C14	0.0484 (9)	0.0384 (8)	0.0293 (8)	−0.0109 (7)	−0.0002 (6)	−0.0039 (6)
C15	0.0562 (10)	0.0441 (9)	0.0446 (10)	−0.0039 (8)	0.0026 (8)	−0.0078 (8)
C16	0.0748 (14)	0.0521 (11)	0.0622 (13)	−0.0078 (10)	0.0193 (11)	−0.0165 (10)
C17	0.1013 (17)	0.0632 (13)	0.0436 (11)	−0.0217 (13)	0.0244 (11)	−0.0197 (10)
C18	0.0886 (15)	0.0665 (13)	0.0284 (9)	−0.0230 (12)	0.0022 (9)	−0.0039 (8)
C19	0.0460 (9)	0.0422 (9)	0.0476 (10)	−0.0026 (7)	−0.0100 (7)	0.0091 (8)
C20	0.0551 (11)	0.0507 (11)	0.0885 (16)	0.0111 (9)	0.0140 (11)	−0.0061 (11)
C21	0.0790 (15)	0.0770 (15)	0.0782 (16)	−0.0006 (12)	0.0305 (12)	−0.0226 (13)
N1	0.0470 (7)	0.0353 (7)	0.0236 (6)	−0.0040 (6)	0.0019 (5)	−0.0024 (5)
N2	0.0454 (7)	0.0323 (6)	0.0254 (6)	0.0009 (5)	−0.0027 (5)	0.0007 (5)
N3	0.0436 (7)	0.0317 (6)	0.0245 (6)	0.0020 (5)	−0.0015 (5)	0.0006 (5)
N4	0.0785 (10)	0.0414 (8)	0.0276 (7)	−0.0099 (7)	−0.0090 (7)	0.0008 (6)
N5	0.0713 (10)	0.0438 (8)	0.0289 (7)	−0.0030 (7)	0.0017 (6)	0.0047 (6)
O1	0.0483 (7)	0.0495 (7)	0.0489 (7)	0.0125 (5)	0.0013 (5)	0.0001 (6)
O2	0.0816 (10)	0.0638 (9)	0.0719 (10)	0.0232 (8)	−0.0223 (8)	0.0150 (7)
O3	0.0609 (7)	0.0542 (7)	0.0291 (6)	−0.0065 (6)	−0.0107 (5)	0.0067 (5)

### Geometric parameters (Å, °)

**Table:**

C1—C2	1.502 (3)	C11—C14	1.442 (2)
C1—H1A	0.9600	C12—O3	1.3895 (18)
C1—H1B	0.9600	C12—C19	1.454 (2)
C1—H1C	0.9600	C13—O3	1.366 (2)
C2—C7	1.383 (2)	C13—C14	1.383 (2)
C2—C3	1.387 (2)	C13—C18	1.383 (2)
C3—C4	1.382 (2)	C14—C15	1.392 (2)
C3—H3	0.9300	C15—C16	1.377 (2)
C4—C5	1.381 (2)	C15—H15	0.9300
C4—N1	1.4177 (18)	C16—C17	1.391 (3)
C5—C6	1.381 (2)	C16—H16	0.9300
C5—H5	0.9300	C17—C18	1.373 (3)
C6—C7	1.376 (3)	C17—H17	0.9300
C6—H6	0.9300	C18—H18	0.9300
C7—H7	0.9300	C19—O2	1.1993 (19)
C8—N2	1.2735 (18)	C19—O1	1.328 (2)
C8—N1	1.3481 (19)	C20—O1	1.440 (2)
C8—N3	1.4287 (17)	C20—C21	1.486 (3)
C9—N5	1.3105 (19)	C20—H20A	0.9700
C9—N3	1.3391 (19)	C20—H20B	0.9700
C9—H9	0.9300	C21—H21A	0.9600
C10—N4	1.303 (2)	C21—H21B	0.9600
C10—N5	1.354 (2)	C21—H21C	0.9600
C10—H10	0.9300	N1—H1	0.862 (17)
C11—C12	1.362 (2)	N3—N4	1.3626 (17)
C11—N2	1.3962 (18)		
			
C2—C1—H1A	109.5	C13—C14—C15	119.15 (15)
C2—C1—H1B	109.5	C13—C14—C11	105.87 (15)
H1A—C1—H1B	109.5	C15—C14—C11	134.89 (15)
C2—C1—H1C	109.5	C16—C15—C14	118.35 (18)
H1A—C1—H1C	109.5	C16—C15—H15	120.8
H1B—C1—H1C	109.5	C14—C15—H15	120.8
C7—C2—C3	117.90 (16)	C15—C16—C17	121.1 (2)
C7—C2—C1	121.85 (16)	C15—C16—H16	119.4
C3—C2—C1	120.23 (16)	C17—C16—H16	119.4
C4—C3—C2	121.35 (15)	C18—C17—C16	121.55 (18)
C4—C3—H3	119.3	C18—C17—H17	119.2
C2—C3—H3	119.3	C16—C17—H17	119.2
C5—C4—C3	119.88 (14)	C17—C18—C13	116.53 (18)
C5—C4—N1	119.40 (14)	C17—C18—H18	121.7
C3—C4—N1	120.64 (13)	C13—C18—H18	121.7
C4—C5—C6	119.21 (16)	O2—C19—O1	124.70 (18)
C4—C5—H5	120.4	O2—C19—C12	124.88 (17)
C6—C5—H5	120.4	O1—C19—C12	110.42 (13)
C7—C6—C5	120.53 (17)	O1—C20—C21	106.89 (16)
C7—C6—H6	119.7	O1—C20—H20A	110.3
C5—C6—H6	119.7	C21—C20—H20A	110.3
C6—C7—C2	121.10 (16)	O1—C20—H20B	110.3
C6—C7—H7	119.5	C21—C20—H20B	110.3
C2—C7—H7	119.5	H20A—C20—H20B	108.6
N2—C8—N1	133.19 (13)	C20—C21—H21A	109.5
N2—C8—N3	115.11 (13)	C20—C21—H21B	109.5
N1—C8—N3	111.70 (12)	H21A—C21—H21B	109.5
N5—C9—N3	110.52 (14)	C20—C21—H21C	109.5
N5—C9—H9	124.7	H21A—C21—H21C	109.5
N3—C9—H9	124.7	H21B—C21—H21C	109.5
N4—C10—N5	115.89 (15)	C8—N1—C4	125.58 (12)
N4—C10—H10	122.1	C8—N1—H1	117.1 (11)
N5—C10—H10	122.1	C4—N1—H1	116.9 (11)
C12—C11—N2	130.93 (14)	C8—N2—C11	123.48 (12)
C12—C11—C14	105.98 (13)	C9—N3—N4	109.44 (12)
N2—C11—C14	122.90 (14)	C9—N3—C8	129.64 (13)
C11—C12—O3	111.34 (14)	N4—N3—C8	120.89 (12)
C11—C12—C19	134.03 (15)	C10—N4—N3	101.83 (13)
O3—C12—C19	114.62 (13)	C9—N5—C10	102.30 (13)
O3—C13—C14	111.09 (14)	C19—O1—C20	117.17 (14)
O3—C13—C18	125.62 (16)	C13—O3—C12	105.68 (12)
C14—C13—C18	123.29 (18)		
			
C7—C2—C3—C4	−1.8 (2)	O3—C12—C19—O2	0.2 (2)
C1—C2—C3—C4	179.70 (16)	C11—C12—C19—O1	1.1 (3)
C2—C3—C4—C5	0.3 (2)	O3—C12—C19—O1	179.71 (13)
C2—C3—C4—N1	177.05 (13)	N2—C8—N1—C4	18.8 (3)
C3—C4—C5—C6	1.4 (2)	N3—C8—N1—C4	−160.66 (13)
N1—C4—C5—C6	−175.39 (15)	C5—C4—N1—C8	−139.07 (16)
C4—C5—C6—C7	−1.5 (3)	C3—C4—N1—C8	44.1 (2)
C5—C6—C7—C2	−0.1 (3)	N1—C8—N2—C11	9.3 (3)
C3—C2—C7—C6	1.7 (3)	N3—C8—N2—C11	−171.20 (13)
C1—C2—C7—C6	−179.82 (18)	C12—C11—N2—C8	62.4 (2)
N2—C11—C12—O3	176.20 (14)	C14—C11—N2—C8	−123.30 (16)
C14—C11—C12—O3	1.20 (17)	N5—C9—N3—N4	−0.78 (19)
N2—C11—C12—C19	−5.2 (3)	N5—C9—N3—C8	−178.69 (14)
C14—C11—C12—C19	179.85 (17)	N2—C8—N3—C9	12.4 (2)
O3—C13—C14—C15	179.20 (14)	N1—C8—N3—C9	−168.00 (15)
C18—C13—C14—C15	0.3 (3)	N2—C8—N3—N4	−165.31 (14)
O3—C13—C14—C11	2.24 (18)	N1—C8—N3—N4	14.28 (19)
C18—C13—C14—C11	−176.69 (16)	N5—C10—N4—N3	−0.7 (2)
C12—C11—C14—C13	−2.05 (17)	C9—N3—N4—C10	0.83 (18)
N2—C11—C14—C13	−177.55 (14)	C8—N3—N4—C10	178.97 (14)
C12—C11—C14—C15	−178.31 (18)	N3—C9—N5—C10	0.34 (19)
N2—C11—C14—C15	6.2 (3)	N4—C10—N5—C9	0.2 (2)
C13—C14—C15—C16	−0.3 (2)	O2—C19—O1—C20	4.3 (3)
C11—C14—C15—C16	175.60 (17)	C12—C19—O1—C20	−175.22 (14)
C14—C15—C16—C17	0.5 (3)	C21—C20—O1—C19	179.39 (16)
C15—C16—C17—C18	−0.7 (3)	C14—C13—O3—C12	−1.52 (17)
C16—C17—C18—C13	0.7 (3)	C18—C13—O3—C12	177.38 (16)
O3—C13—C18—C17	−179.23 (16)	C11—C12—O3—C13	0.14 (17)
C14—C13—C18—C17	−0.5 (3)	C19—C12—O3—C13	−178.79 (13)
C11—C12—C19—O2	−178.42 (18)		

### Hydrogen-bond geometry (Å, °)

**Table:**

*D*—H···*A*	*D*—H	H···*A*	*D*···*A*	*D*—H···*A*
C10—H10···O2^i^	0.93	2.43	3.271 (2)	150
N1—H1···N5^ii^	0.862 (17)	2.250 (17)	3.0755 (19)	160.3 (15)

Symmetry codes: (i) *x*−1/2, −*y*+1/2, *z*−1/2; (ii) −*x*+1/2, *y*−1/2, −*z*+3/2.
